# A yeast-based *in vivo* assay complements *in vitro* and *in silico* approaches to characterize sterol-binding by *Saccharomyces cerevisiae* Lam/Ltc proteins

**DOI:** 10.1016/j.jbc.2025.110663

**Published:** 2025-09-01

**Authors:** Ola El Atab, Barkha Gupta, Zhu Han, Rabih Darwiche, Stéphanie Cottier, Jiri Stribny, Roger Schneiter

**Affiliations:** Department of Biology, University of Fribourg, Fribourg, Switzerland

**Keywords:** lipid transfer proteins, Lam/Ltc proteins, sterol binding, phosphatidylserine, *in vivo* assay, *Saccharomyces cerevisiae*, StARkin domain, microscale thermophoresis, membrane contact sites

## Abstract

Cellular membranes maintain distinct lipid compositions, with sterols enriched in the plasma membrane despite their synthesis in the endoplasmic reticulum (ER). This distribution relies on vesicular and nonvesicular transport, the latter facilitated by lipid transfer proteins (LTPs) at membrane contact sites. In yeast, the Lam/Ltc family of LTPs is critical for sterol transport. To characterize their sterol-binding properties in a cellular context, we developed a yeast-based *in vivo* assay using *Saccharomyces cerevisiae*. Here, sterol-binding proteins, fused to signal sequences, extract and export radiolabeled cholesterol from the luminal compartment of the secretory pathway to the culture medium, offering a qualitative measure of binding capacity. We demonstrate that yeast Lam/Ltc proteins (Ysp1, Ysp2, Lam4, Lam5, and Lam6) and their StARkin domains efficiently extract sterols, complementing the activity of known LTPs (Pry1, NPC2, Osh4, and STARD1). *In vitro* microscale thermophoresis and *in silico* docking confirmed low micromolar to nanomolar sterol affinities. Notably, Lam6 binds phosphatidylserine through its GRAM domain, with synergistic binding to membranes containing both phosphatidylserine and ergosterol. Although limited by its qualitative nature and luminal specificity, the *in vivo* sterol binding assay complements *in vitro* methods, providing a robust tool to study LTPs in a cellular environment. These findings enhance our understanding of Lam/Ltc protein function and highlight the assay’s potential for characterizing known and orphan LTPs.

Lipids serve as essential building blocks for membranes, influencing critical properties such as fluidity, curvature, and protein recruitment, which affect cellular processes including signaling, trafficking, and metabolism. They are highly diverse in their chemical structure and differ in their subcellular distribution (1). Exchange of lipids within and between intracellular membranes is mediated by vesicular and nonvesicular lipid transport (2). Nonvesicular transport is thought to occur mainly at membrane contact sites (MCSs), *i.e.*, at sites where two organellar membranes are closely apposed to each other (3, 4).

Lipid transport at MCSs is facilitated by proteins that function either to shuttle lipids between the two membranes as is the case for lipid transfer proteins (LTPs), or that form lipid-conductive bridges (5, 6). Based on their lipid-binding specificities, LTPs can transfer sterols, phospholipids, or sphingolipids and their precursors (7, 8). LTPs typically contain one or more lipid-binding domains to extract the lipid from the membrane, shuttle it through the aqueous space and release it into the membrane of the acceptor compartment. These proteins are characterized by the presence of an internal cavity that is frequently capped by a flexible lid.

In yeast, three different families of sterol-binding and transport proteins have been identified: oxysterol-binding protein (OSBP) and its related protein homologs (oxysterol-binding protein related-proteins [ORPs]), LTPs anchored at MCSs (Lam/Ltc), which contain steroidogenic acute regulatory protein related lipid transfer (StART/VASt) domains found in the StARkin superfamily, and CAP proteins (cysteine-rich secretory proteins, antigen 5, pathogenesis-related protein 1), a class of secreted glycoproteins associated with immune regulation in plants and animals (5, 9, 10).

ORPs/OSBP are conserved across the eukaryotic kingdom and are implicated in many cellular processes including cell signaling, vesicular trafficking, lipid metabolism, nonvesicular sterol transport, and formation of MCSs (11, 12). These proteins are characterized by the presence of an OSBP-related ligand-binding domain (ORD), which contains a hydrophobic pocket capable of accommodating sterols and phospholipids (13, 14). In the model organism *Saccharomyces cerevisiae*, this protein family is represented by seven Osh proteins that share an essential function in vegetative growth as the combined deletion of all seven genes is lethal (15). Osh4/Kes1 is one of the best characterized and most abundant member of the yeast ORPs (16, 17). It binds sterols and phosphatidylinositol-4-phosphate (PI4P) to transport sterols against a concentration gradient (18, 19, 20, 21, 22).

Humans possess 15 different StART domain containing proteins, among which the founding member of the StARkin superfamily, StAR/STARD1, which transports cholesterol into mitochondria in steroidogenic cells, and the well-characterized STARD4 protein, which transports cholesterol to the endoplasmic reticulum (ER) and the endocytic recycling compartment (23, 24, 25). A protein family distantly related to StART proteins was identified in yeast, plants, and metazoans (26, 27, 28, 29). In yeast, these Lam/Ltc proteins form three distinct groups, each of which contains two paralogs: Ysp1/Sip3, Ysp2/Lam4, and Lam5/Lam6. These proteins possess an N-terminal glucosyltransferase, Rab-like GTPase activator and myotubularin (GRAM) domain, which has structural similarity to the pleckstrin homology domain, either one or two StarD/VASt/StARkin lipid-binding domains, and they are anchored to the ER membrane through predicted transmembrane domains (TMDs) in their C-terminal part (27, 28, 30). Ysp1, Ysp2, Sip3, and Lam4 play an important role in sterol transfer between the ER and the plasma membrane (PM), whereas Lam5 and Lam6 have a role in sterol transfer between the ER and mitochondria (27, 28, 31, 32). In metazoans, these proteins include three Aster proteins (Aster-A, B, C), also known as GRAM domain containing proteins (GRAMDs; GRAMD1a, 1b, and 1c) (29, 33, 34). The StARkin domain of these proteins adopts a truncated version of the α/β helix-grip fold found in StART proteins. It forms a cup-like structure that provides a hydrophobic cavity to bind sterol in exchange for water and is covered by a lid (29, 30, 35, 36, 37, 38).

Proteins belonging to the third class of sterol-binding proteins, the CAP superfamily, termed pathogen-related in yeast (Pry) were identified as promoting the secretion of acetylated sterols (9, 39). The yeast genome encodes for three different Pry proteins, which are characterized by the presence of a unique conserved CAP domain (40). Pry1 and Pry2 are secreted glycoproteins whereas *PRY3* encodes a glycosylphosphatidylinositol-anchored cell wall protein (41, 42). Pry1 and Pry2 share a redundant function in the secretion of cholesteryl acetate, as their combined deletion completely blocks the secretion of sterols (9, 39).

Although significant progress has been made in understanding the structure and function of these sterol-binding proteins, a limitation in the field has been the reliance on *in vitro* assays that may not fully recapitulate the complexity of the cellular environments. We hypothesized that secreted sterol-binding proteins could extract and export sterols from membranes of the secretory pathway in an engineered yeast system, thereby providing a qualitative *in vivo* measure of their sterol-binding capacity *in vivo*.

In this study, we aimed to characterize the lipid-binding properties of yeast Lam/Ltc proteins both *in vitro* and *in vivo*. To complement established *in vitro* approaches, we developed a yeast-based *in vivo* sterol export assay, validated using well-characterized sterol transfer proteins: the pathogenesis-related protein 1 (Pry1), Niemann-Pick type C2 (NPC2) protein, oxysterol-binding protein homolog 4 (Osh4), and steroidogenic acute regulatory protein 1 (STARD1) (13, 43, 44). Our results demonstrate that these proteins, along with yeast Lam/Ltc family members (Ysp1, Ysp2, Sip3, Lam4, Lam5, and Lam6), promote the export of radiolabeled cholesterol when secreted from yeast cells, indicating sterol extraction from the luminal compartment of the secretory pathway. To corroborate and extend these findings, selected Lam proteins and their StARkin domains were purified and assessed for cholesterol binding *via* microscale thermophoresis (MST), revealing low micromolar to nanomolar affinities (13, 39, 45, 46). Liposome flotation assays further showed that Lam6 associates with membranes containing ergosterol or phosphatidylserine (PS), with synergistic effects when both lipids are present. Collectively, these data highlight the multifaceted lipid interactions of Lam/Ltc proteins and underscore the complementary value of *in vivo* and *in vitro* methods for evaluating LTP function in cellular contexts.

## Results

### Validation of the *in vivo* sterol-binding and export assay

**Figure 1:**
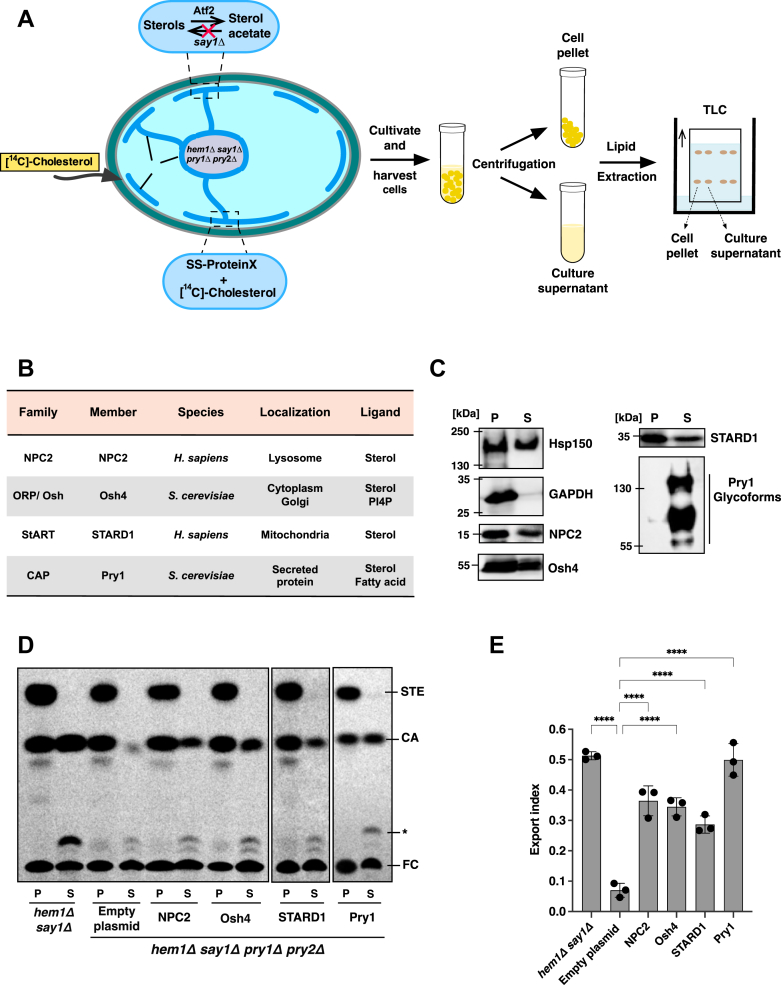
**NPC2, Osh4, STARD1, and Pry1 bind and export sterols *in vivo*.***A*, outline of the experimental design. Yeast cells possess a sterol acetylation/deacetylation cycle, which is localized in the ER and mediated by two enzymes: Atf2 is required for acetylation of free sterols whereas Say1 is required for its deacetylation. Simultaneous deletion of Say1, and the sterol exporting CAP family proteins Pry1 and Pry2, results in the accumulation of acetylated sterols within the ER. Expression of a putative sterol-binding protein of interest containing an N-terminal signal sequence (SS-ProteinX) directs the protein into the secretory pathway, where it can bind and then export the radiolabeled cholesteryl acetate into the culture medium. The deletion of *HEM1* blocks intracellular sterol synthesis and promotes uptake of radiolabeled cholesterol. After labeling of cells with [^14^C]-cholesterol, cells are collected, centrifuged, and lipids are extracted from both the cell pellet and culture supernatant fractions. Lipids are then separated by thin-layer chromatography (TLC), visualized by phosphor imaging, and quantified. *B,* summary of sterol binding proteins tested in the *in vivo* assay. Gene names, host species, the subcellular localization, and major lipid ligands of the proteins that were tested in the yeast based *in vivo* assay are indicated. *C*, expression and secretion of sterol binding protein in yeast cells. NPC2, Osh4, STARD1, and Pry1 were fused to an N-terminal signal sequence and C terminally tagged with a HA-epitope. Proteins were precipitated from both the cell pellet and culture supernatant and analyzed by Western blotting. NPC2, Osh4, STARD1, and Pry1 were all expressed and secreted into the culture media as was the secreted heat shock protein, Hsp150. GAPDH, an intracellular enzyme, on the other hand, was only detected in the cell pellet. *D*, quadruple mutant cells (*hem1Δ say1Δ pry1Δ pry2Δ)* containing either an empty plasmid or a plasmid-borne copy of NPC2, Osh4, STARD1, or Pry1 were cultivated in the presence of [^14^C]-cholesterol. Lipids were extracted from the cell pellet (P) and the culture supernatant (S), separated by TLC and visualized by phosphorimaging. The position of free cholesterol (FC), cholesteryl acetate (CA), steryl esters (STE), and an unidentified lipid (∗) are indicated to the *right*. *E*, quantification of cholesteryl acetate export. The export index was calculated as the ratio of extracellular CA to total CA (intracellular + extracellular). Data shown in *panelE* represent the mean ± SD of three independent experiments, and statistical significance is indicated: ∗∗∗∗*p* < 0.0001 (one-way ANOVA with Bonferroni's *post hoc* test). ER, endoplasmic reticulum; NPC2, Niemann-Pick type C2; Pry, pathogen-related in yeast; STARD1, steroidogenic acute regulatory protein 1.

The *in vivo* sterol binding and export assay developed in this study leverages a genetically modified yeast strain with four strategic gene deletions (Fig. 1*A*). Deletion of *HEM1* blocks intracellular sterol synthesis and renders cells sterol auxotrophs, enabling precise radiolabeling through the addition of exogenous [^14^C]-cholesterol (47). Deletion of the deacetylating enzyme *SAY1* prevents sterol deacetylation in the ER, while deletion of the two redundant sterol-transporting proteins Pry1 and Pry2 inhibits the export of acetylated cholesterol (39, 48). This combination of mutations results in the accumulation of radiolabeled [^14^C]-cholesterol and its acetylated derivative in the ER of the quadruple mutant strain (*hem1Δ say1Δ pry1Δ pry2Δ*).

To assess sterol-binding and export capacity, the protein of interest is fused to an N-terminal signal sequence derived from either the prepro-alpha-factor pheromone or from Pry1, directing it into the secretory pathway. There, the protein can bind radiolabeled cholesterol and/or its acetylated derivative and export it from the cell (39, 48, 49). Lipids are then extracted from both the cell pellet and culture supernatant and analyzed by thin-layer chromatography (TLC) (Fig. 1*A*). The relative proportion of radiolabeled cholesteryl acetate exported from cells is quantified, normalized to its total level, and plotted as an export index.

### NPC2, Osh4, and STARD1 demonstrate sterol-binding and export activity *in vivo*

To validate the yeast-based *in vivo* export assay, we selected three well-characterized sterol-binding proteins: human NPC2, yeast Osh4, and human STARD1 (Fig. 1*B*) (13, 43, 44). We first confirmed proper expression and secretion of these proteins by tagging them with a C-terminal hemagglutinin (HA) epitope and fusing them to N-terminal signal sequences (prepro-alpha-factor for NPC2; Pry1 signal sequence for Osh4 and STARD1).

Western blot analysis of both cell pellets and culture supernatants confirmed successful expression and secretion of all proteins (Fig. 1*C*). NPC2, Osh4, and STARD1 were detected at their expected molecular weights (17 kDa, 52 kDa, and 35 kDa, respectively), corresponding to their predicted molecular masses of 16.5 kDa, 49.4 kDa, and 31.9 kDa. Pry1 appeared as a triplet of bands (55 kDa to >120 kDa) due to glycosylation (39). The secreted glycoprotein Hsp150 and the cytoplasmic GAPDH (glyceraldehyde-3-phosphate dehydrogenase) served as controls for secretion efficiency and cell integrity, respectively (50).

Having confirmed protein expression and secretion, we tested their functionality in the sterol export assay. Quadruple mutant cells expressing these proteins were labeled with [^14^C]-cholesterol, and lipids were extracted from both cell pellets and culture supernatants for TLC analysis. The double mutant strain (*hem1Δ say1Δ*) exported cholesteryl acetate due to the presence of WT copies of *PRY1* and *PRY2*. However, simultaneous deletion of *PRY1* and *PRY2* blocked sterol export in the quadruple mutant (*hem1Δ say1Δ pry1Δ pry2Δ*). This export block was overcome by the expression of a plasmid-borne copy of *PRY1* or by expression of signal sequence-containing versions of NPC2, Osh4, or STARD1, all of which resulted in cholesteryl acetate export (Fig. 1, *D* and *E*).

These results demonstrate that NPC2, Osh4, and STARD1 can functionally complement the absence of Pry1 and Pry2 proteins, despite being targeted to the ER lumen—a nonphysiological compartment for these cytoplasmic proteins—validating the *in vivo* export assay as a tool to monitor sterol binding of cytoplasmic proteins. Furthermore, these findings indicate that NPC2, Osh4, and STARD1 can bind not only free cholesterol but also its acetylated derivative, and that they can extract and solubilize sterols from membranes within the secretory pathway (39, 48).

### *In vitro* binding studies confirm sterol-binding properties of NPC2, Osh4, and STARD1

**Figure 2:**
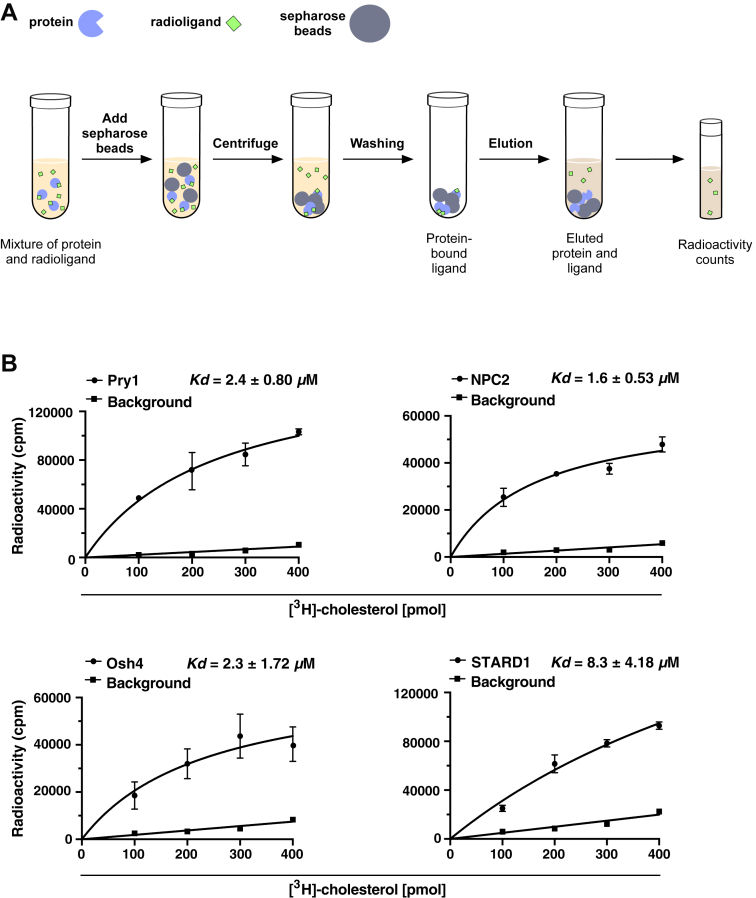
***In vitro* cholesterol binding by Pry1, NPC2, Osh4, and STARD1 as assessed by radioligand binding assay.***A*, *in vitro* cholesterol binding as assessed by a radioligand binding assay. Schematic representation of the workflow of the *in vitro* lipid binding assay. Purified proteins (*blue*, 100 pmol), different concentrations of radiolabeled [^3^H]-cholesterol (*green*, 0–400 pmol) are incubated together to allow binding. Proteins were separated from unbound ligand by adsorption to an ion exchange matrix *(gray*), followed by washing steps. The protein-bound ligand is then eluted and quantified by liquid scintillation counting. Nonspecific binding is assessed in parallel by performing the assay in the absence of protein. *B*, cholesterol binding by Pry1, NPC2, Osh4, and STARD1. Binding by Pry1 and Osh4 was assessed at pH 7.5, that of STARD1 at pH 8.5, and NPC2 binding affinity was determined at pH 5.5. The dissociation constant (*K*_*d*_) was deduced from the binding curve: Pry1 bound cholesterol with a *K*_*d*_ of 2.4 μM, NPC2 with a *K*_*d*_ of 1.6 μM, Osh4 with a *K*_*d*_ of 2.3 μM, and STARD1 bound cholesterol with a *K*_*d*_ of 8.3 μM. Data shown in *panelB* represent the mean ± SD of three independent experiments. NPC2, Niemann-Pick type C2; Pry, pathogen-related in yeast; STARD1, steroidogenic acute regulatory protein 1.

To validate these *in vivo* findings with independent *in vitro* approaches, Pry1, NPC2, Osh4, and STARD1 were expressed as C terminally polyhistidine-tagged proteins in *Escherichia coli* and purified using Ni^2+^-NTA agarose beads. Cholesterol binding of these proteins was then assessed using a radioligand binding assay with a constant protein concentration (100 pmol) and increasing concentrations of [^3^H]-cholesterol (0–400 pmol). Protein-bound radioligand was separated from unbound ligand by absorption to an ion-exchange matrix (13, 51) (Fig. 2*A*). Consistent with the *in vivo* results, all proteins bound cholesterol with micromolar affinity: Pry1 with a dissociation constant (*K*_*d*_) of 2.4 μM, NPC2 with a *K*_*d*_ of 1.6 μM, Osh4 with a *K*_*d*_ of 2.3 μM, and STARD1 with a *K*_*d*_ of 8.3 μM (Fig. 2*B*).

**Figure 3:**
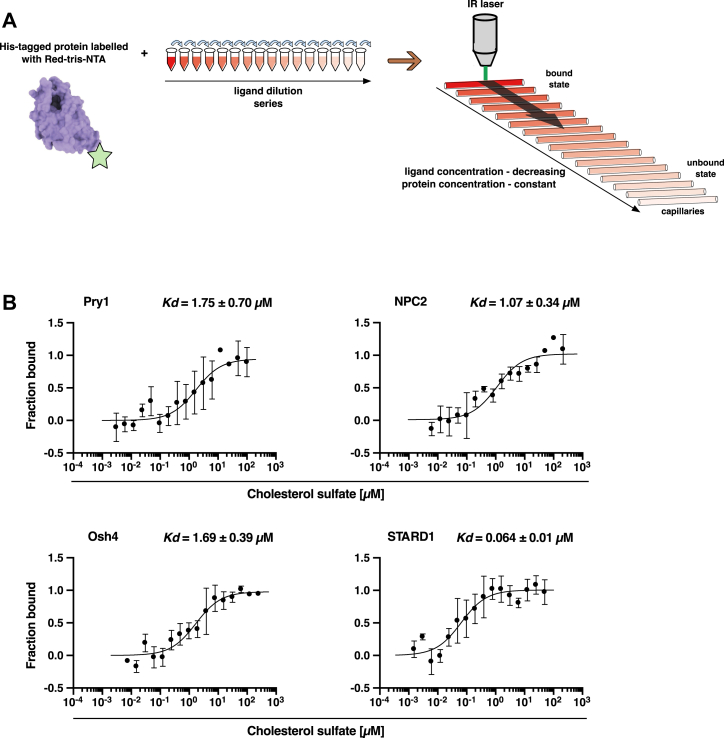
**Binding of cholesterol sulfate by Pry1, NPC2, Osh4, and STARD1 monitored by microscale thermophoresis.***A*, scheme illustrating the principle of the MST-based ligand binding assay. This polyhistidine-tagged protein is site-specific fluorescently labeled (RED-tris-NTA, *green star*). The labeled protein is incubated with varying concentrations of ligand, loaded into glass capillaries, the capillaries are locally heated with an infrared (IR) laser, generating a temperature gradient, and the rate of temperature-induced diffusion of the ligand-bound protein was recorded and plotted as a function of ligand concentration. *B*, sterol binding by Pry1, NPC2, Osh4, and STARD1 measured by MST. The indicated protein (50 nM) was incubated with different concentrations of cholesterol sulfate (1.5 nM-100 μM), and sterol binding was monitored by MST. Binding by Pry1, Osh4 was assessed at pH 7.5, STARD1 at pH 8.5, and NPC2 binding affinity was determined at pH 5.5. Dissociation constants (*K*_*d*_) were derived from the binding curves: Pry1 (1.75 μM), NPC2 (1.07 μM), Osh4 (1.69 μM), and STARD1 (0.064 μM). Data shown in *panelB* represent the mean ± SD of three independent experiments. MST, microscale thermophoresis; NPC2, Niemann-Pick type C2; Pry, pathogen-related in yeast; STARD1, steroidogenic acute regulatory protein 1.

To corroborate these findings with an independent method, we employed MST, in which proteins were noncovalently labeled with a fluorescent dye specific for the C-terminal polyhistidine tag (52). The labeled proteins were incubated with varying concentrations of cholesterol sulfate, a water-soluble cholesterol analog used to overcome the limited solubility of native cholesterol, and binding was assessed by measuring differences in temperature-dependent mobility of the fluorescent protein (45, 46, 53) (Fig. 3*A*).

MST analysis revealed saturable binding for all tested proteins, with Pry1 binding cholesterol sulfate with a *K*_*d*_ of 1.75 μM, NPC2 with a *K*_*d*_ of 1.07 μM, Osh4 with a *K*_*d*_ of 1.69 μM, and STARD1 with a *K*_*d*_ of 0.064 μM (Fig. 3*B*). The MST measurements typically yielded lower dissociation constants compared to the radioligand assay (Table 1), likely due to differences in experimental conditions, including the ligands used (cholesterol *versus* cholesterol sulfate), binding buffers, and the separation step required in the radioligand binding assay. We consider the MST readout more robust as it combines more data points, does not require physical separation of assay components, and employs a more water-soluble lipidic ligand, reducing potential limitations due to ligand solubility.

**Table 1:** Comparative analysis of binding affinities between radioligand and MST assays

Protein	Radioligand binding assay *K*_*d*_ [μM]	MST *K*_*d*_ [μM]
Pry1	2.4	1.75
NPC2	1.6	1.07
Osh4	2.3	1.69
STARD1	8.3	0.064

### Lam/Ltc family proteins export cholesteryl acetate *in vivo*

**Figure 4:**
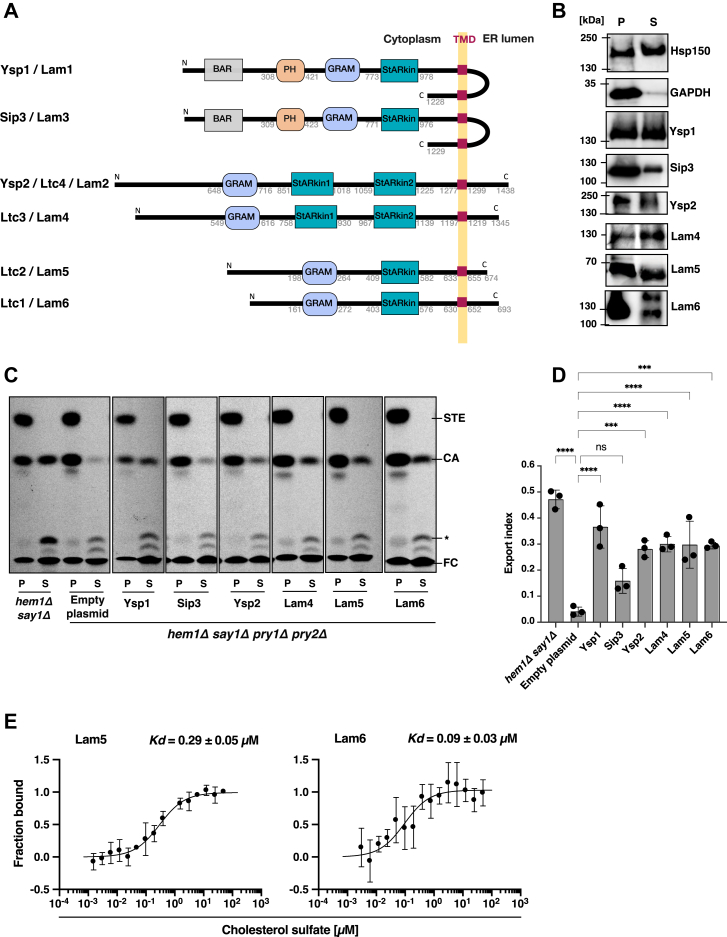
**Ysp1, Ysp2, Lam4, Lam5, and Lam6 bind and export cholesterol *in vivo*, Lam5 and Lam6 bind sterols *in vitro.****A*, illustration of the domain structure of the yeast Lam/Ltc family members. BAR, pleckstrin homology (PH), glucosyltransferase, Rab-like GTPase activator and myotubularin (GRAM), and the predicted sterol binding StARkin domains are represented as *filled boxes*. All six Lam/Ltc family members have a predicted transmembrane domain (TMD) that anchors them to the ER membrane, and a short C-terminal part. *B*, expression and secretion of soluble domains of Lam/Ltc proteins in yeast cells. Lam/Ltc proteins fused to an N-terminal signal sequence and containing a C-terminal HA-tag were expressed in yeast cells. Proteins were precipitated from both the cell pellet and culture supernatant and analyzed by western blotting. Ysp1, Sip3, Ysp2, Lam4, Lam5, and Lam6 were expressed and efficiently secreted into the culture media. The heat-shock protein Hsp150 and the cytosolic GAPDH were included to monitor the efficiency of the secretory pathway and cell integrity. The position of molecular weight standards is shown on the left-hand side of each blot. *C* – *D*, export of cholesteryl acetate by Lam/Ltc proteins into the culture supernatant was examined using a quadruple mutant yeast strain lacking the endogenous sterol exporting proteins Pry1 and Pry2 (*hem1Δ say1Δ pry1Δ pry2Δ*). Export index values show that Ysp1, Ysp2, Lam4, Lam5, and Lam6 efficiently exported sterol *in vivo*, whereas Sip3 showed reduced levels of export. *E*, Lam5 and Lam6 bind sterols *in vitro*. Purified proteins were fluorescently labeled and binding to cholesterol sulfate was determined by MST. The dissociation constant, *K*_*d*_ was determined from the binding curve, Lam5, *K*_*d*_ of 0.29 μM, Lam6, *K*_*d*_ of 0.09 μM. Data shown in *panelD* represent the mean ± SD of three independent experiments and statistical significance is indicated: ∗∗∗*p* < 0.001, ∗∗∗∗*p* < 0.0001 (one-way ANOVA with Bonferroni's *post hoc* test). ns, not significant. ER, endoplasmic reticulum; HA, hemagglutinin; MST, microscale thermophoresis; Pry, pathogen-related in yeast.

To characterize cholesterol binding and export by proteins of the Lam/Ltc family, we expressed the soluble portions of these proteins (lacking C-terminal transmembrane domains, ΔTMD) fused to signal sequences derived from either Pry1 (for Sip3) or prepro-alpha-factor pheromone (for Ysp1, Ysp2, Lam4, Lam5, and Lam6) (Fig. 4*A*). The constructs included Sip3 (1–1060 aa), Ysp1 (1–1020 aa), Ysp2 (1–1271 aa), Lam4 (1–1196 aa), Lam5 (1–633 aa), and Lam6 (1–621 aa), each C terminally tagged with a HA epitope and expressed in the quadruple mutant strain.

Western blot analysis confirmed expression and secretion of all proteins: Sip3 (121.3 kDa), Ysp1 (129.5 kDa), Ysp2 (151.6 kDa), Lam4 (133.4 kDa), Lam5 (72.5 kDa), and Lam6 (80.7 kDa) (Fig. 4*B*). Cytosolic GAPDH and secreted Hsp150 served as controls for cell integrity and secretion efficiency, respectively.

When cells expressing these proteins were labeled with [^14^C]-cholesterol, five of the six Lam/Ltc proteins (Ysp1, Ysp2, Lam4, Lam5, and Lam6) exported cholesteryl acetate into the culture supernatant, demonstrating their ability to functionally complement the absence of Pry proteins. Sip3 showed reduced export, possibly due to poor secretion and/or aberrant folding within the luminal compartment (Fig. 4, *C* and *D*).

To corroborate these *in vivo* findings, we expressed soluble versions (ΔTMD) of Lam5 and Lam6 as polyhistidine-tagged proteins and assessed their sterol binding capacity using MST. Both proteins bound cholesterol sulfate with low micromolar affinity: Lam5 with a *K*_*d*_ of 0.29 μM and Lam6 with a *K*_*d*_ of 0.09 μM (Fig. 4*E*), confirming their direct sterol-binding capability.

### StARkin domains of Lam family members exhibit differential sterol-binding properties

**Figure 5:**
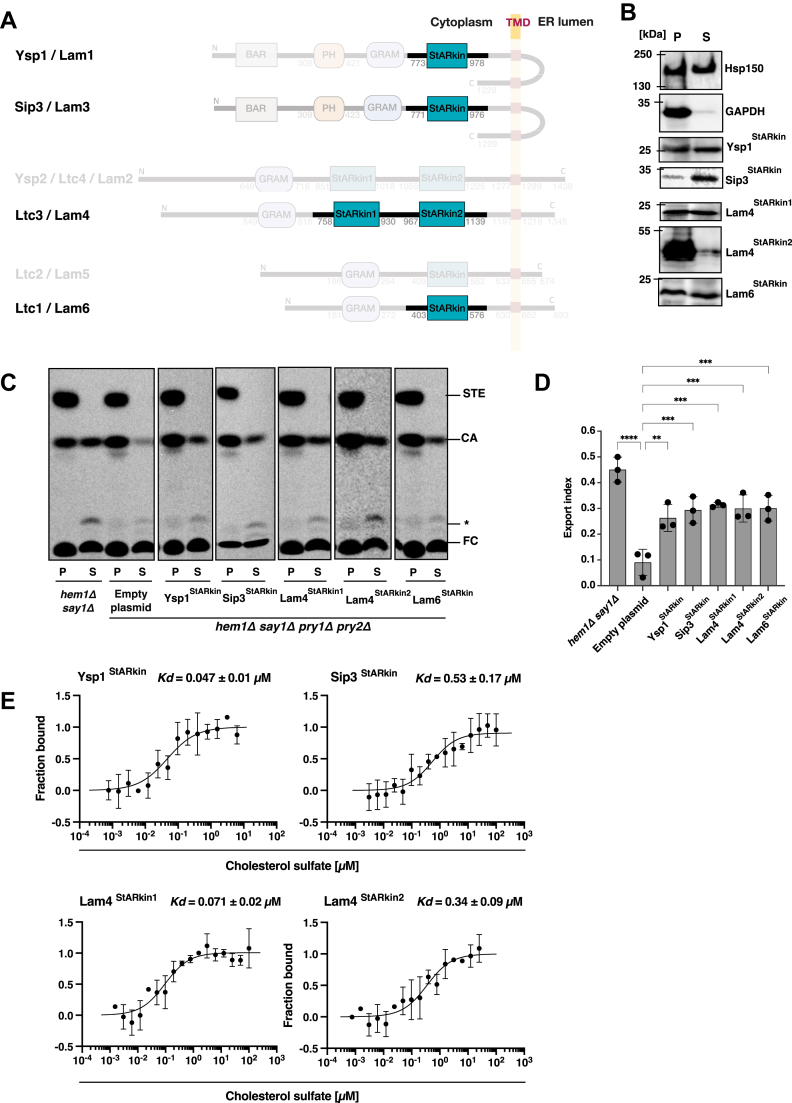
**The StARkin domains of Ysp1, Sip3, Lam4, and Lam6 bind cholesterol.***A*, illustration of the domain structure of the yeast Lam/Ltc family members with the StARkin domains highlighted as filled boxes. *B*, expression and secretion of soluble StARkin domains of Lam/Ltc proteins in yeast cells. The StARkin domains of Ysp1 (amino acids 765–978; 26.9 kDa), Sip3 (amino acids 733–1002, 32.5 kDa), as well as the two StARkin domains of Lam4 (StARkin1, amino acids 758–930, 20.3 kDa, and StARkin2, amino acids 967–1139, 34.6 kDa), and that of Lam6 (amino acids 401–579, 22 kDa) were fused to an N-terminal signal sequence from prepro-alpha-factor and appended with a C-terminal HA-tag. Plasmids were transformed into yeast cells and proteins were precipitated from both the cell pellet and culture supernatant and analyzed by western blotting. Ysp1^StARkin^, Sip3^StARkin^, Lam4^StARkin1^, Lam4^StARkin2^, and Lam6^StARkin^ were expressed and, except for Lam4^StARkin2^, efficiently secreted into the culture medium. The heat-shock protein Hsp150 and the cytosolic GAPDH were included to monitor the efficiency of the secretory pathway and cell integrity. The position of molecular weight standards is shown on the left-hand side of each blot. *C* – *D*, export of cholesteryl acetate by the StARkin domains of Lam/Ltc proteins was examined using a quadruple mutant yeast strain (*hem1Δ say1Δ pry1Δ pry2Δ*). The export index indicates that Ysp1^StARkin^, Sip3^StARkin^, Lam4^StARkin1^, Lam4^StARkin2^, and Lam6^StARkin^ exported sterol *in vivo* with significantly higher rates than cells containing an empty plasmid. *E*, Ysp1^StARkin^, Sip3^StARkin^, Lam4^StARkin1^, and Lam4^StARkin2^ bind sterols *in vitro*. Purified StARkin domains were fluorescently labeled and binding to cholesterol sulfate was determined by MST. The dissociation constant, *K*_*d*_ was determined from the binding curve, Ysp1^StARkin^ (0.047 μM), Lam4^StARkin1^ (0.071 μM), Lam4^StARkin2^ (0.34 μM), and Sip3^StARkin^ (0.53 μM). Data shown in panel *D* represent the mean ± SD of three independent experiments and statistical significance is indicated: ∗∗*p* < 0.01, ∗∗∗*p* < 0.001, ∗∗∗∗*p* < 0.0001 (one-way ANOVA with Bonferroni’s *post hoc* test). HA, hemagglutinin; MST, microscale thermophoresis.

Given that the soluble domain of Ysp1 exported sterols while its paralog Sip3 did not, we investigated whether this discrepancy might result from improper folding of Sip3 in the ER lumen. We therefore expressed individual StARkin domains from these proteins: Ysp1^StARkin^ (765–978 aa, 26.9 kDa), Sip3^StARkin^ (733–1002 aa, 32.5 kDa), both StARkin domains of Lam4 (StARkin1, 758–930 aa, 20.3 kDa; StARkin2, 967–1139 aa, 34.6 kDa), and Lam6^StARkin^ (401–579 aa, 22 kDa) (Fig. 5*A*).

Western blot analysis showed that four of the five domains (Ysp1^StARkin^, Sip3^StARkin^, Lam4^StARkin1^, and Lam6^StARkin^) were efficiently expressed and secreted, while Lam4^StARkin2^ was highly expressed but poorly secreted (Fig. 5*B*). Remarkably, all five domains promoted cholesteryl acetate export when expressed in the quadruple mutant background (Fig. 5, *C* and *D*), including the Sip3^StARkin^ domain. This suggests that the isolated domain is functional, while the full-length protein may be misfolded or otherwise impaired.

To further characterize these domains, we expressed and purified Ysp1^StARkin^, Sip3^StARkin^, Lam4^StARkin1^, and Lam4^StARkin2^ from bacterial cells and assessed their sterol-binding affinities using MST. All four domains bound cholesterol sulfate, with Ysp1^StARkin^ and Lam4^StARkin1^ showing the highest affinities (*K*_*d*_ of 0.047 μM and 0.071 μM, respectively). Lam4^StARkin2^ exhibited approximately 5-fold lower affinity (*K*_*d*_ of 0.34 μM) compared to Lam4^StARkin1^, while Sip3^StARkin^ (*K*_*d*_ of 0.53 μM) showed 11-fold lower affinity than its paralog Ysp1^StARkin^ (Fig. 5*E*). These measured binding affinities are consistent with previously reported values, such as a *K*_*d*_ of 0.5 μM for the StARkin domain of Lam4 (27).

These findings reveal substantial variation in sterol-binding affinities among different StARkin domains, which may account for the apparent redundancy of these proteins and their function under different physiological conditions. Interestingly, these differences in *in vitro* binding affinities were not directly reflected in the *in vivo* export capacities as quantified by the export index (Fig. 5, *C* and *D*).

### Molecular docking predicts high-affinity interactions between ergosterol and StARkin domains

**Figure 6:**
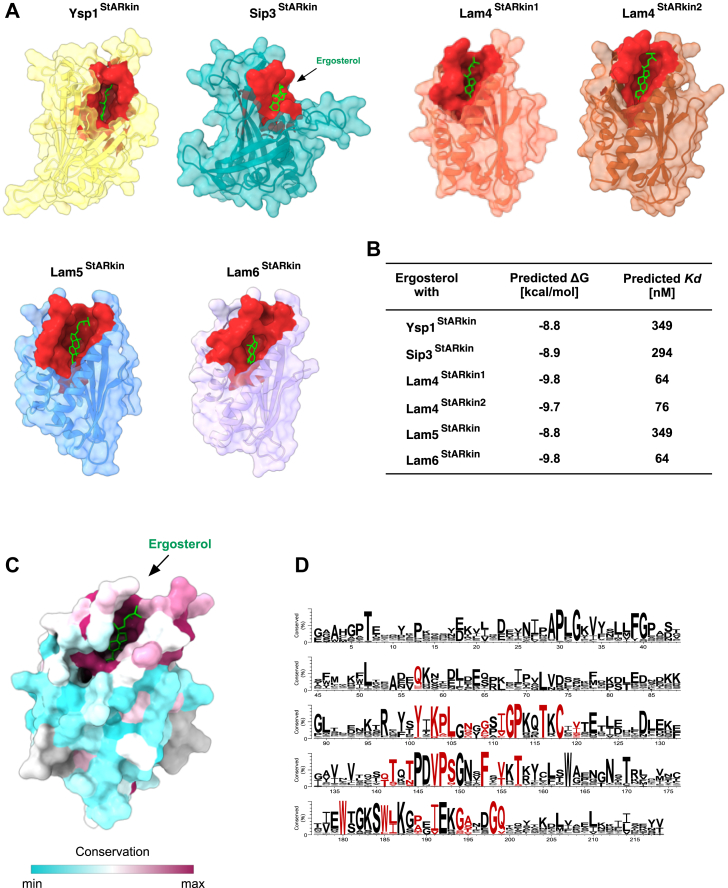
**Evolutionary conserved amino acids within the binding cavities of Lam family proteins mediate ergosterol interaction.***A*, protein-ligand docking simulations were conducted using AutoDock Vina to analyze interactions and predict binding affinities between ergosterol (*green*) and the StARkin domains of Ysp1 (*yellow*), Sip3 (*teal*), Lam4 (StARkin1, tomato; StARkin2, *sienna*), Lam5 (*blue*), and Lam6 (*purple*). Protein structures used for *in silico* docking were predicted using AlphaFold2 and illustrated by employing a blend of ribbon and surface representations. In each StARkin domain, ergosterol is predicted to bind in a head-down orientation within the hydrophobic binding pocket. The upper region of the binding cavity representing the ergosterol binding site is highlighted in *red*. *B*, the table illustrates the predicted interaction between ergosterol and the StARkin domains of Lam proteins in terms of free energy (ΔG; in kcal/mol) and binding affinity (*K*_*d*_; in nM) for each interaction, as predicted by AutoDock Vina. *C*, Surface representation of the Lam4 StARkin domain 1, color coded according to sequence conservation determined by the ConSurf server. Conservation scores were calculated from a multiple sequence alignment of 300 LAM orthologs. The conservation scale ranges from *cyan* (low conservation) to *maroon* (high conservation). *D*, multiple sequence alignment of StARkin domains of LAM orthologs, obtained *via* the Consurf server. Amino acids lining the ergosterol binding site are highlighted in *red*.

To better understand the molecular basis for the observed differences in binding affinities, we conducted *in silico* docking experiments using AutoDock Vina to predict interactions between ergosterol and various StARkin domains (54, 55). Since experimentally determined structures were only available for the StARkin domains of Ysp2 and Lam4 (30, 36, 37), we used AlphaFold to predict structures for the StARkin domains of Ysp1, Sip3, Lam4 (both domains), Lam5, and Lam6 (56). When overlaid with experimentally determined structures, the AlphaFold-predicted structures of Lam4's two StARkin domains showed excellent agreement (RMSD values of 0.448 Å and 0.410 Å, respectively), validating the computational approach (Fig. 6). The StARkin domain adopts a helix-grip fold characterized by curved β-sheets forming a hydrophobic cavity capable of accommodating a single sterol molecule, complemented by a flexible lid formed by the Ω1 loop between the β2 and β3 strands.

These docking experiments revealed that ergosterol binds in the upper part of the cavity in all tested StARkin domains, consistent with crystal structures of sterol-bound forms of Ysp2 and Lam4 StARkin domains (Fig. 6*A*). Ergosterol adopts a head-down orientation with its 3-hydroxyl group facing the bottom of the hydrophobic cavity, a binding mode common among sterol transfer proteins, including some Osh family members (13, 57).

The predicted interactions between ergosterol and StARkin domains showed high affinity across all domains tested, with low ΔG values and binding affinities (*K*_*d*_) as summarized in Figure 6*B*. The predicted *K*_*d*_ showed that Lam4^StARkin1^ and Lam6^StARkin^ have the highest affinity to ergosterol. Analysis of conserved residues of all Lams StARkin domains revealed high conservation of amino acids lining the hydrophobic pocket (Fig. 6, *C* and *D*). Although these results highlight the structural similarity among Lam StARkin domains and their similar sterol binding modes, the *in silico* docking experiments did not identify specific structural elements accounting for the observed differences in binding affinities between the two StARkin domains of Lam4.

### Lam6 shows enhanced binding to membranes containing both phosphatidylserine and ergosterol

**Figure 7:**
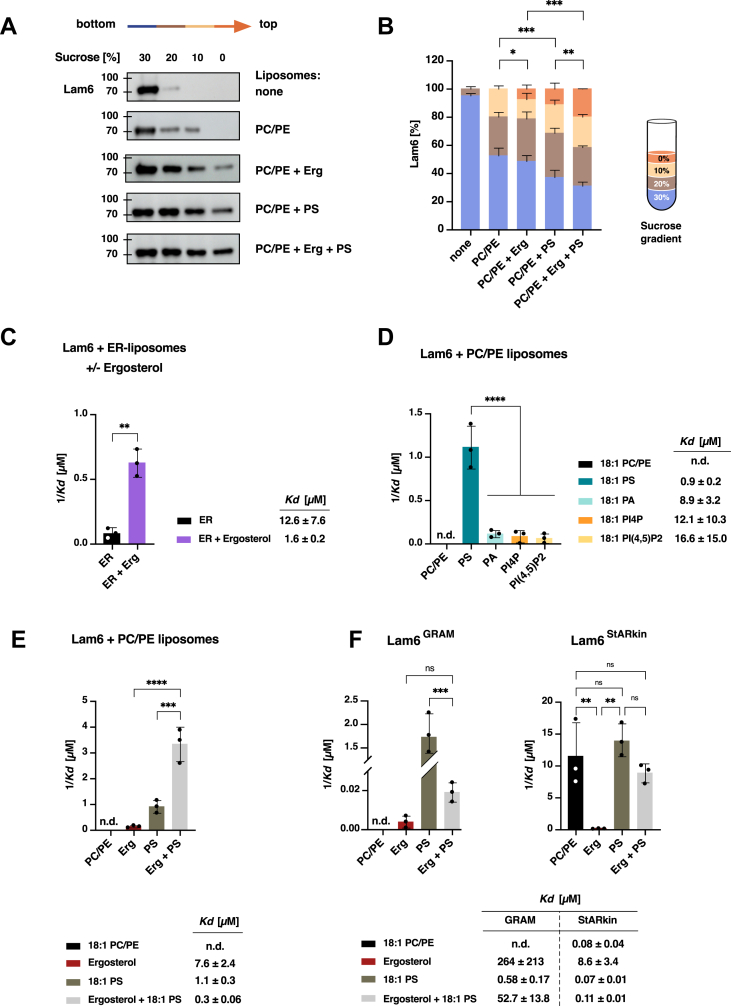
**Lam6 shows increased binding affinity to liposomes containing both ergosterol and phosphatidylserine.***A*, binding of Lam6 to liposomes composed of DOPC (80 mol%) and DOPE (20 mol%) with or without supplementation of ergosterol (5 mol%) or DOPS (5 mol%) or both. Following incubation of Lam6 with liposomes, the samples were subjected to sucrose density gradient centrifugation, that enabled separation of proteins bound to the buoyant liposomes from unbound proteins. All fractions, from top to bottom of the density gradient were analyzed by Western blotting; ‘none’ indicates no liposomes were added in the flotation assay. *B*, quantification of Lam6 distribution in different fractions of the sucrose gradient. The signals from individual fractions were quantified and the statistical significance of Lam6 recruitment to the liposomes top fraction (*orange*) is indicated. *C*, Lam6 binding to ER-liposomes consisting of DOPC (53 mol%), DOPE (23 mol%), DOPS (8 mol%), DOPA (5 mol%), and SoyPI (11 mol%) supplemented or not with ergosterol (5 mol%), as measured by MST. *D*, Lam6 binding to liposomes (DOPC/DOPE; 80/20 mol%) was assessed with the addition of negatively charged lipids, either DOPS (5 mol%), DOPA (5 mol%), PI4P (5 mol%) or PI(4,5)P_2_ (5 mol%). *E*, Lam6 binding to liposomes composed of DOPC/DOPE (80/20 mol%) was evaluated in the presence of ergosterol (5 mol%) or DOPS (5 mol%) or a combination of both (each at 5 mol%). *F*, binding of the GRAM domain of Lam6 to liposomes is stimulated by PS. Liposome (DOPC/DOPE; 80/20 mol %) binding by the GRAM or StARkin domain of Lam6 was assessed in the presence of ergosterol (5 mol %), DOPS (5 mol %), or both ergosterol and DOPS (each at 5 mol%) by MST. *K*_*d*_ values determined in *panelsC*, *D*, *E*, and *F* are plotted as reciprocal values. All data represent the mean ± SD of three independent experiments. *Asterisks* denote statistical significance: ∗*p* < 0.05; ∗∗*p* < 0.01, ∗∗∗*p* < 0.001, ∗∗∗∗*p* < 0.0001 (data shown in *panelsB*, *D*, *E*, and *F* were analyzed by one-way ANOVA with Tukey’s *post hoc* test, while *panelC* was analyzed by unpaired *t* test, two-tailed). ns, not significant; n.d., not detected. DOPA, 1,2-dioleoyl-sn-glycero-3-phosphate; GRAM, GTPase activator and myotubularin; MST, microscale thermophoresis; PI4P, phosphatidylinositol-4-phosphate.

The human orthologs of Lam proteins, the Aster proteins (particularly Aster-A/GRAMD1a and Aster-B/GRAMD1b), as well as yeast Ysp2 and Lam4, have been shown to bind to liposomes containing anionic phospholipids, especially PS (29, 30, 58, 59, 60). To investigate the lipid binding specificities of Lam6, one of the best characterized members of the Lam/Ltc family, we employed a liposome flotation assay (28, 31). In this assay, bacterially purified and his-tagged soluble Lam6 (ΔTMD) was incubated with liposomes of varying lipid compositions, followed by sucrose density gradient centrifugation and visualization of Lam6 binding through western blotting. Remarkably, Lam6 exhibited significantly enhanced binding to liposomes containing either low concentrations of PS (DOPS; 5 mol%) or ergosterol (5 mol%) compared to basic phosphatidylcholine (PC)/phosphatidylethanolamine (PE) liposomes (DOPC/DOPE; 80/20 mol%). Moreover, the simultaneous presence of both PS and ergosterol in liposomes resulted in synergistically enhanced Lam6 binding compared to liposomes containing either lipid alone (Figs. 7, *A*, *B* and S1). These findings suggest that PS may serve as an additional lipid ligand for Lam6 alongside ergosterol, with even low concentrations of either lipid being sufficient to stimulate Lam6 binding to membranes.

To quantitatively assess these interactions with greater sensitivity, we employed MST to measure Lam6 (ΔTMD) binding to liposome of various compositions. First, we prepared complex liposomes mimicking ER membrane lipid composition (DOPC/DOPE/DOPS/DOPA/SoyPI; 53/23/8/5/11 mol%). The addition of ergosterol (5 mol%) to these ER-like liposomes dramatically increased binding affinity by 7-fold (*K*_*d*_ of 12.6 ± 7.6 μM vs. *K*_*d*_ of 1.6 ± 0.2 μM), indicating that ergosterol significantly enhances Lam6 binding (Fig. 7*C*).

To test whether a specific phospholipid enhances liposome binding affinity, we prepared simplified liposomes containing the two major ER membrane phospholipids, PC and PE (DOPC/DOPE; 80/20 mol%), supplemented with individual phospholipids: PS (DOPS; 5 mol%), PA (1,2-dioleoyl-sn-glycero-3-phosphate [DOPA]; 5 mol%), PI4P (5 mol%), or PI(4,5)P2 (5 mol%). Aster-B/GRAMD1b and GRAMD2a have previously been shown to associate with PI4P and PI(4,5)P_2_-containing membranes, so these phosphoinositides were included in the analysis (33, 37). MST measurements revealed that Lam6 interacted most strongly with PS-containing liposomes (*K*_*d*_ of 0.9 ± 0.2 μM) compared to liposomes containing other phospholipids (Fig. 7*D*). These results demonstrate that Lam6 selectively binds PS rather than merely recognizing negatively charged phospholipids. This specificity likely accounts for the affinity observed with ER-liposomes containing PS and ergosterol.

To further quantify the synergistic effect observed in flotation assays when both PS and ergosterol were present, we prepared PC/PE liposomes (DOPC/DOPE; 80/20 mol%) with or without ergosterol, PS, or both lipids. MST analysis confirmed a striking synergistic enhancement of Lam6 binding when both PS and ergosterol were present (*K*_*d*_ 0.3 ± 0.06 μM), compared to liposomes containing either ergosterol alone (*K*_*d*_ 7.6 ± 2.4 μM) or PS alone (*K*_*d*_ 1.1 ± 0.3 μM) (Fig. 7*E*).

To test whether the GRAM domain of Lam6 could function as a coincidence detector recognizing both PS and ergosterol simultaneously, as observed with the GRAM domain of Aster-B/GRAM1b, we assessed liposome binding by isolated GRAM and StARkin domains (58, 60). The purified GRAM domain of Lam6 exhibited dramatically increased binding affinity toward liposomes containing PS (*K*_*d*_ 0.58 ± 0.17 μM) compared to basic PC/PE liposomes or those supplemented with ergosterol (Fig. 7*F*). Intriguingly, this enhanced PS-dependent binding was substantially reduced in the presence of ergosterol, suggesting that ergosterol may hinder PS accessibility to the GRAM domain.

In contrast, the StARkin domain of Lam6 showed highest binding affinity toward PC/PE liposomes regardless of whether they contained PS or PS plus ergosterol (*K*_*d*_ 0.11 ± 0.01 μM). Unexpectedly, binding was strongly decreased when ergosterol alone was incorporated into the liposomes (*K*_*d*_ 8.6 ± 3.4 μM) (Fig. 7*F*). Collectively, these results reveal that Lam6 exhibits high affinity binding not only to ergosterol but also to specific phospholipids, primarily PS, most likely mediated through its GRAM domain.

## Discussion

The development of *in vivo* assays to investigate lipid-protein interactions and their roles in lipid homeostasis is crucial yet challenging. Although many powerful tools have been established for *in vitro* studies, relatively few *in vivo* assays are available (61, 62, 63, 64, 65, 66). In this study, we developed an *in vivo* lipid binding assay using *S. cerevisiae* that allows the characterization of lipid–protein interactions and lipid transport in a cellular context. It is important to emphasize that the *in vivo* assay described here serves as a qualitative readout that reveals whether a protein can bind and export sterols. It does not serve as a quantitative readout or reveal potential binding affinities, because the protein needs to be targeted to the luminal compartment of the secretory pathway, an oxidative environment in which silent glycosylation sites might get recognized, potentially affecting the protein's sterol binding properties. Expression of the protein of interest under the *ADH1* promoter ensured consistent moderate levels across constructs, but results might vary with native promoters or different signal sequences (67). A key advantage of this approach is that it relies on proteins extracting and solubilizing sterols from a biological membrane rather than from a low complexity *in vitro* system such as aqueous solutions or liposomes.

This assay employs a yeast quadruple mutant strain that is unable to synthesize or export cholesterol. By expressing proteins of interest with appropriate signal sequences that direct them into the secretory pathway, the export of protein-bound lipids from cells can be monitored. The results of this assay were validated using complementary *in vitro* cholesterol-binding assays with purified proteins. To establish the reliability of the *in vivo* lipid-binding assay, we tested well-characterized sterol-binding proteins: NPC2, Osh4, and STARD1. The successful export of cholesteryl acetate upon expression and ER luminal targeting of any of these proteins confirmed the functionality of the assay (Fig. 1). Further *in vitro* cholesterol-binding assays, using both radioligand-based methods and MST revealed that these proteins bind cholesterol with nanomolar to low micromolar affinity (Figure 2, Figure 3 and 3).

Having validated the approach, the *in vivo* lipid-binding assay was applied to characterize the sterol-binding properties of yeast LAM family proteins. Among the six yeast LAM proteins tested, five (Ysp1, Ysp2, Lam4, Lam5, and Lam6) exported significant levels of cholesteryl acetate, demonstrating their ability to bind cholesterol and transport it into the extracellular space (Fig. 4). Consistent with this *in vivo* activity, soluble ΔTMD versions of Lam5 and Lam6 bound cholesterol sulfate with low nanomolar affinity *in vitro* as measured by MST (Fig. 4*E*).

Interestingly, while the soluble version of Sip3 exported only low levels of sterols in the *in vivo* assay, its isolated StARkin domain was sufficient for sterol export. This observation prompted us to investigate whether the StARkin domains alone from various LAM family proteins could support sterol binding and export. Indeed, expression of the StARkin domains from Ysp1, Sip3, Lam4, and Lam6 was sufficient for sterol export *in vivo*. Furthermore, both StARkin domains present in Lam4 bound cholesterol sulfate *in vitro*, albeit with different affinities (Fig. 5), suggesting potential functional differences within these domains.

To understand the molecular basis for the different affinities observed between the two StARkin domains of Lam4, *in silico* ligand docking experiments using models based on the known structures of Ysp2 and Lam4 were performed (Fig. 6) (30, 36, 37). The StARkin domain is characterized by a hydrophobic cavity formed by a central α-helix and curved β-sheets, complemented by a flexible lid composed of the Ω1 loop. Previous studies have highlighted the significance of this Ω1 loop in sterol binding, with molecular dynamics simulations suggesting its involvement in the entry and release of sterol ligands (68, 69, 70).

**Figure 8:**
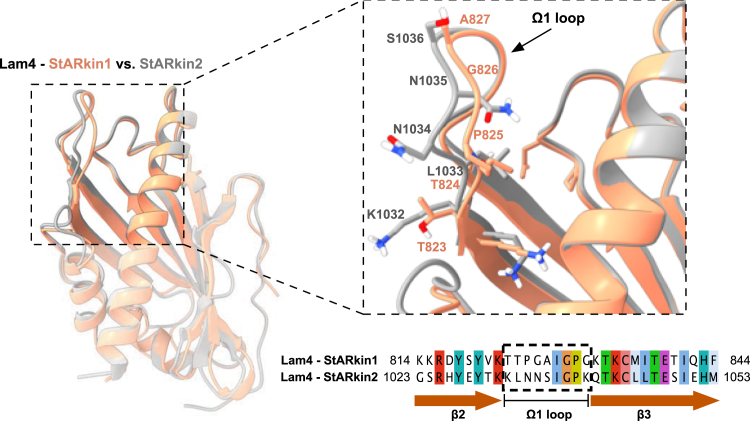
**Structural and sequence comparison of the StARkin domain 1 and domain 2 of Lam4.***A*, structural superimposition of the StARkin domain 1 (*orange*) and domain 2 (*gray*) of Lam4 is shown in the *upper left-handpanel*. A close-up view of the Ω1 loop present between the β2 and β3 strands, with the amino acids indicated is shown in the *upper right-handpanel*. A sequence alignment of the two StARkin domains of Lam4 generated using MAFFT and visualized with Jalview is shown in the *lower right-hand panel* of the figure. Residues within the Ω1 loop are highlighted for comparison.

Structural comparison of the two StARkin domains of Lam4 revealed notable differences in the Ω1 loop within an otherwise conserved cavity structure (Fig. 8). These structural disparities likely contribute to the observed differences in ligand affinity between the two sterol-binding domains. Such variations in binding properties may reflect evolutionary adaptations to distinct cellular contexts or sterol concentrations, potentially explaining the apparent redundancy of these domains within a single protein.

Beyond sterol binding, we investigated the membrane interaction properties of LAM family proteins. The human orthologs of Lam proteins (Aster proteins, particularly Aster-A/GRAMD1a and Aster-B/GRAMD1b), as well as yeast Ysp2 and Lam4 have been shown to bind to liposomes containing anionic phospholipids, especially PS (29, 30, 58, 59, 60). Given that Lam6 is one of the best-characterized members of the Lam family—known to localize to various membrane contact sites (ER-mitochondria, vacuole-mitochondria, and nuclear-vacuolar junctions) and to interact strongly with subunits of the ERMES complex—we investigated whether soluble Lam6 (ΔTMD) would exhibit similar preferences for membranes containing negatively charged phospholipids (28, 31).

Lam6 indeed displayed increased affinity for PS-containing liposomes in a flotation assay (Figs. 7, *A*, *B* and S1). Remarkably, when tested with a complex lipid composition resembling that of the ER membrane (containing 8 mol% PS), the inclusion of low concentrations of ergosterol (5 mol%) enhanced Lam6 binding affinity by more than 7-fold (Fig. 7*C*). This binding was specific to PS and not observed with other negatively charged phospholipids such as phosphatidic acid or phosphoinositides (Fig. 7*D*).

When tested with either ER-like liposomes or simple PC/PE liposomes, the simultaneous presence of PS and ergosterol resulted in a synergistic increase in Lam6 binding affinity (Fig. 7*E*). This observation is consistent with the notion that the full-length protein functions as a coincidence detector for both lipids, similar to what has been observed for the GRAM domain of Aster-B/GRAMD1b (58, 60). However, unlike the GRAM domain of Aster-B/GRAMD1b, the isolated GRAM domain of Lam6 appeared to recognize PS only, and its affinity for liposomes was greatly decreased in the presence of ergosterol (Fig. 7*F*).

These findings suggest that despite their structural relationship, the GRAM domains of Lam proteins and Aster-B/GRAMD1b harbor different lipid binding specificities and may confer distinct functions to their respective full-length proteins. Interestingly, the GRAM domain of Lam6 alone is sufficient for targeting to ERMES, suggesting that this targeting may be mediated by recognition of PS-rich membrane domains (30). In contrast, PS enriched in the inner leaflet of the PM is important for Aster/GRAMD1-mediated uptake and transport of cholesterol to the ER and endocytic compartments (29, 58, 59, 60, 71, 72).

The StARkin domain of Lam6, on the other hand, showed high binding affinity to PC/PE liposomes in general, but this affinity was strongly reduced in the presence of ergosterol (Fig. 7*F*). Thus, for both the GRAM and StARkin domains alone, the presence of ergosterol in liposomes reduced their binding affinities by approximately 78 to 90 fold. Given that sterols in phospholipid membranes increase phospholipid packing, these results suggest that the lipid binding specificities and affinities of these domains are strongly affected by sterol-mediated bilayer condensation (73, 74, 75, 76, 77).

Although the *in vivo* sterol export assay provides a valuable qualitative measure of sterol-binding capacity in a cellular context, it has several limitations. Primarily, the assay targets proteins to the ER lumen, which represents a nonphysiological compartment for many cytoplasmic LTPs and a low-sterol environment compared to the PM, potentially limiting its ability to fully recapitulate natural lipid extraction dynamics. In addition, reliance on radiolabeled cholesterol restricts its applicability to other lipids, such as phospholipids or nonacetylated sterols; future enhancements could incorporate nonradioactive detection methods or mass spectrometry to identify a broader range of bound lipids. The *in vitro* assays, including MST and liposome flotation, employ nonphysiological conditions, such as cholesterol sulfate, detergents like Triton X-100, or simplified liposome compositions, that may influence binding affinities and not fully mimic cellular membrane complexity. Despite these constraints, the assay complements traditional *in vitro* approaches, offering insights into LTP function within a living cell and paving the way for characterizing orphan lipid-binding proteins.

In conclusion, this study demonstrates that yeast Lam/Ltc family proteins can bind sterols *in vitro* and *in silico* and effectively export them *in vivo*. Differences in structural elements, such as within the Ω1 loop, likely underlie variations in ligand affinity and suggest functional specialization in distinct cellular contexts. In addition, Lam6 displays selective binding to PS-rich membranes, an interaction modulated by the presence of ergosterol. These findings highlight the functional diversity and potential adaptability of Lam/Ltc family proteins in lipid binding and underscore the value of combining *in vivo* and *in vitro* approaches to study membrane association. In this regard, the sterol-binding and export assay presented here establishes a versatile tool to monitor sterol binding and transport by diverse lipid-binding protein families *in vivo*. Although this study focused on sterols, future work will explore whether similar *in vivo* approaches can monitor binding to other lipid classes, such as phospholipids or ceramides. This may reveal *in vivo* lipid-binding properties of other lipid-binding domains, including the GRAM domain, offering new insights into the mechanisms of cellular lipid transport and homeostasis.

## Experimental procedures

### *S. cerevisiae* strains and growth conditions

*S. cerevisiae* double mutant (*hem1Δ say1Δ*) and quadruple mutant cells (*hem1Δ say1Δ pry1Δ pry2Δ*) were generated using PCR-based gene deletion cassettes and a marker rescue strategy (78). Double mutant strains were cultivated in YPD medium (1% bacto yeast extract, 2% bacto peptone and 2% glucose; US Biological). Quadruple mutants were cultivated in synthetic complete medium (0.67% yeast nitrogen base without amino acids (US Biological), 0.73 g/l amino acids and 2% glucose). The genotype of strains employed in this study is given in Supporting Information, Table S1.

To compensate for the heme deficiency, cells were grown in media supplemented with 10 μg/ml delta-aminolevulinic acid during transformation, or with 20 μg/ml cholesterol (Sigma Chemical Co) dissolved in Tween-80 during the export assay (39, 48).

Plasmids encoding proteins of interest were constructed by fusing their open reading frame to a signal sequence from either Pry1 or the prepro-alpha-factor pheromone and a C-terminal HA-tag. Expression was driven by the constitutive *ADH1*-promoter (79). Genes were PCR-amplified from *S. cerevisiae* genomic DNA or synthesized as codon-optimized cDNAs (NPC2 and STARD1; GenScript) and inserted into plasmid pRS416 (80). A list of plasmids used in this study is given in Supporting Information, Table S2.

### *In vivo* lipid export assay

The sterol export assay, which allows for the examination of sterol acetylation and secretion into the culture supernatant, was essentially performed as previously described (39, 48). *S. cerevisiae* mutants deficient in heme biosynthesis (*hem1Δ*) and lacking the sterol deacetylase enzyme Say1 (*say1Δ*) were cultivated overnight in the presence of unlabeled cholesterol/Tween-80. On the second day, cells were collected by centrifugation, washed twice with synthetic complete medium and diluted to an *A*_600_ of 1 into fresh medium containing 0.025 μCi/ml [^14^C]-cholesterol (0.1 mCi/ml, specific activity 55 mCi/mmol; American Radiolabeled Chemicals, Inc).

After overnight growth, cells were washed again and cultivated for an additional day in nonradiolabeled cholesterol-containing media. Cells were then centrifuged, lysed by disruption with glass beads, and lipids were extracted from the cell pellet and the culture supernatant using chloroform/methanol (1:1, v/v) (81). Extracted radiolabeled lipids were quantified and volumes corresponding to 5000 cpm (counts per minute) were dried and resuspended in chloroform/methanol. Lipids were separated by TLC on silica gel 60 plates (Merck) using the solvent system petroleum ether/diethyl ether/acetic acid (70:30:2, v/v/v).

TLC plates were then exposed to phosphorimaging screens and radiolabeled lipids were visualized and quantified using a phosphorimager (GE Healthcare). The cholesterol export index was calculated as the ratio of extracellular cholesteryl acetate to the sum of intracellular and extracellular cholesteryl acetate. Export assays were performed in triplicate and the export index is given as the mean ± SD of three independent experiments.

### Protein secretion analysis and western blotting

Proteins were tagged with a HA-epitope (YPYDVPDYA) to analyze their expression and secretion. Proteins were extracted from three *A*_600_ units of cells using an NaOH lysis method followed by precipitation with 10% trichloroacetic acid (82). To analyze proteins in the culture supernatant, proteins from 20 ml of medium were precipitated with 10% trichloroacetic acid, washed with acetone, solubilized in sample buffer and analyzed by SDS-PAGE.

Western blotting was performed using a rat anti-HA antibody (rat, 1:2000, Roche #11867423001), or a c-Myc monoclonal antibody (mouse, 1:5,000, Invitrogen #13-2500) to detect Hsp150-myc. As secondary antibodies, horseradish peroxidase conjugate goat anti-rat IgG antibody (1:10,000, Merck #AP136P), or horseradish peroxidase conjugated goat anti-mouse IgG (1:10,000, Bio-Rad #1706516) were used.

### Protein expression and purification under native conditions

For protein expression and purification from *E. coli*, DNA encoding proteins of interest was cloned into XhoI/BamHI or XhoI/NcoI restriction sites of pET22b (Novagen, Merck), which contains a PelB signal sequence to direct the secretion of expressed proteins into the periplasmic space. For LAM proteins, the transmembrane domains were excluded and only the soluble domains were expressed. Plasmids were transformed into *E. coli* BL21 and proteins were expressed with a C-terminal polyhistidine-tag.

For protein purification, different induction strategies were used. Expression of Pry1, Osh4, Ysp1^StARkin^, Lam5, Lam4^StARkin1^, and Lam4^StARkin2^, was induced with lactose overnight at 24 °C. STARD1 was purified after induction with 0.5 mM IPTG for 4 h at 37 °C, followed by overnight freezing of cells at −20 °C. Expression of Lam6 was induced with 0.4 mM IPTG for 6 h at 24 °C.

Cells were collected, lysed, and incubated with nickel-nitrilotriacetic acid (Ni^2+^-NTA) beads (Qiagen) as per the manufacturer's instructions. Beads were washed, loaded onto a Ni^2+^-NTA column (Qiagen) and proteins were eluted with 60 mM NaH_2_PO_4_ (pH 8.0), 300 mM NaCl and 300 mM imidazole. Protein concentrations were determined by the Lowry assay using the Folin reagent and bovine serum albumin as standard (83).

### Protein purification under denaturing conditions

The *NPC2* gene was cloned into XhoI/NcoI restriction sites of pET22b (Novagen, Merck). Protein expression was induced with 0.5 mM IPTG for 6 h at 30 °C. NPC2 aggregated in inclusion bodies and therefore was purified under denaturing conditions. After induction, cells were collected, lysed, and washed twice with 50 mM Tris (pH 8.5), 500 mM NaCl.

The pellet was then resuspended in solubilization buffer (50 mM Tris–HCl (pH 8.8), 7 M urea, 2 mM thiourea, 4% CHAPS, and 5% glycerol), sonicated for 10 min and incubated on a shaker at 55 °C, with 1300 rpm for 30 min. The urea was then diluted to 4 M with binding buffer (60 mM NaH_2_PO_4_ (pH 8.8), 300 mM NaCl). Affinity purification of the renatured protein was performed as described above for the native proteins.

### Protein expression and purification from *Lactococcus lactis*

DNA encoding proteins of interest was cloned into NcoI/HindIII restriction sites of pNZ8048 vector and plasmids were transformed into *L. lactis* DML1 strain. Proteins were expressed with a C-terminal polyhistidine-tag. *L. lactis* cells were grown under anaerobic conditions at 28 °C in M17 broth (Merck Millipore) supplemented with 1% glucose and with 10 μg/ml chloramphenicol for pNZ8048 plasmid maintenance.

For protein purification, expression of Sip3^StARkin^, Lam6^StARkin^, and Lam6^GRAM^ under the control of the pNisA promoter was induced with 2.5 μg/liter nisin during the log phase at *A*_600_ ∼0.5, followed by a 2-h culture before harvesting. Cells were collected, lysed, and incubated with nickel-nitrilotriacetic acid beads (Qiagen) according to the manufacturer's instructions. Beads were washed, loaded onto a Ni^2+^-NTA column (Qiagen) and proteins were eluted with 20 mM Tris (pH 7.5), 300 mM NaCl, and 300 mM imidazole.

### *In vitro* lipid binding assay

The *in vitro* radioligand binding assay was performed essentially as previously described (13, 39). Purified proteins (100 pmol) were incubated with 100 to 400 pmol of [^3^H]-cholesterol (1 mCi/ml, specific activity 50 Ci/mmol; American Radiolabeled Chemicals, Inc), in binding buffer for 1 h at 30 °C. The pH of the binding buffer was adjusted based on the isoelectric point of the protein: for Pry1 and Osh4, 20 mM Tris (pH 7.5), 30 mM NaCl; for STARD1, the same buffer at pH 8.5; and for NPC2, 20 mM potassium acetate (pH 5.5), 30 mM NaCl.

Proteins were then separated from unbound ligand by adsorption to SP Sepharose (GE Healthcare) beads for NPC2, and to Q Sepharose beads (GE Healthcare) beads for all other proteins. Beads were washed and proteins were eluted with binding buffer containing 1 M NaCl. Protein-bound radioligand was quantified by scintillation counting. Ion exchange beads without protein were used to define nonspecific binding. Data were analyzed using Prism software (GraphPad) and are presented as the mean ± SD from three independent experiments.

### Liposome preparation

The following lipids were used for liposome preparation, and unless otherwise noted, were purchased from Avanti Polar Lipids: DOPA (18:1 #840875), 1,2-dioleoyl-sn-glycero-3-phosphocholine (18:1 DOPC, #850375), 1,2-dioleoyl-sn-glycero-3-phosphoethanolamine (18:1 DOPE, #850725), 1,2-dioleoyl-sn-glycero-3-phospho-L-serine sodium salt (18:1 DOPS, #840035), L-α-phosphatidylinositol sodium salt (Soy PI, #840044), 1,2-dioleoyl-sn-glycro-3-phospho-(1′-myo-inositol-4′-phosphate) ammonium salt (18:1 PI4P, #850115), 1,2-dioleoyl-sn-glycro-3-phospho-(1′-myo-inositol-4′,5′-bisphosphate) ammonium salt (18:1 PI(4,5)P2, #850155), Ergosterol (Merck, #E6510).

Lipids were dried from chloroform stock solutions using a rotary evaporator or under a gentle nitrogen gas stream. The dried lipid film was resuspended in 1 ml liposome buffer (25 mM Tris (pH 7.5), 50 mM NaCl) to a final concentration of 2 mM phospholipids. The phospholipid suspension was then subjected to ten freeze-thaw cycles, alternating between liquid nitrogen and a 55 °C water bath.

The resulting multilamellar liposomes were extruded nineteen times through a polycarbonate filter of 0.8 μm pore size to generate large unilamellar vesicles. The size distribution of the large unilamellar vesicles was determined by dynamic light scattering (NanoLab 3D, LS Instruments AG), and liposomes showed a homogeneous size distribution with a mean diameter between 90 and 200 nm.

### Liposome flotation assay

Purified Lam6 was incubated with liposomes for 1 h at room temperature, and then gently mixed with an equal volume of 60% (w/v) sucrose solution in liposome buffer to obtain a final sucrose concentration of 30%. This mixture was overlaid with two volumes of 20% sucrose solution, two volumes of 10% sucrose solution, and one volume of liposome buffer. Samples were centrifuged at 177,000*g* for 1 h at 20 °C. Four fractions were collected from the top of the gradient, and the distribution of Lam6 was analyzed by western blotting. Signal intensities were quantified using ImageJ software.

### Microscale thermophoresis

MST experiments were performed to assess protein-cholesterol sulfate and protein-liposome interactions on a Monolith NT.115 (NanoTemper Technologies). Purified proteins were fluorescently labeled using the RED-tris-NTA His tag protein labeling kit (NanoTemper Technologies) (52). Labeled proteins (100 nM) were mixed with a serial dilution of cholesterol sulfate (cholesterol sulfate, #700016; Avanti Polar Lipids), or liposomes, prepared in binding buffer (20 mM Tris (pH 7.5), 30 mM NaCl, 0.05% Triton X-100). Binding by Pry1, Osh4, and the StARkin domains was assessed at pH 7.5, STARD1 at pH 8.5, and NPC2 binding affinity was determined at pH 5.5.

The mixtures, containing 50 nM of labeled protein and a dilution of cholesterol sulfate or liposomes, were incubated for 20 min at 30 °C. Samples were centrifuged for 5 min at 15,000*g* at 4 °C and loaded into standard MST capillaries. The dissociation constant *K*_*d*_ was determined by plotting the fraction of ligand-bound protein against the logarithm of the ligand concentrations, using Prism software (GraphPad).

### *In silico* ligand docking

Docking of ergosterol to the StARkin domains of Lam proteins was assessed using AutoDock Vina (54, 55). The predicted docking sites between ergosterol and StARkin domains were analyzed and visualized using UCSF Chimera X (84, 85).

## Data availability

All data are contained within the manuscript.

## Supporting information

This article contains Supporting Informations (48, 86).

## Conflict of interest

The authors declare that they have no conflicts of interest with the contents of this article.
