# Supplementary material for: A yeast-based in vivo assay complements in vitro and in silico approaches to characterize sterol-binding by Saccharomyces cerevisiae Lam/Ltc proteins
Source: J Biol Chem. 2025 Sep 1;301(10):110663. doi: 10.1016/j.jbc.2025.110663 (PMC12493155; doi:10.1016/j.jbc.2025.110663)
Supplement: Supporting Figure and Tables [file mmc1.docx]

**Supporting Information**

**Supporting Figures**

**Figure S1. Lam6 shows increased binding affinity to liposomes containing both ergosterol and phosphatidylserine.**

Quantification of Lam6 distribution in different fractions of the sucrose gradient. The signals from individual fractions were quantified and the statistical significance of Lam6 recruitment to the liposomes top fraction (orange) is indicated. Asterisks denote statistical significance: **p* < 0.05; ***p* < 0.01, ****p* < 0.001, *****p* < 0.0001 (data shown were analyzed by one-way ANOVA with Tukey’s post hoc test).

**Supporting Tables**

**Table S1.** *Saccharomyces cerevisiae* strains used in this study

| **Name** | ***Relevant Genotype*** | | **Source** |
| --- | --- | --- | --- |
| RSY6382  RSY1853  RSY6761  RSY4733 | *BY4741; MATa his3∆1 leu2∆0 met15∆0 ura3∆0*  *MATα his3∆1 leu2∆0 lys2∆0 ura3∆0 say1::KanMX hem1::LEU2*  *MATa pry1::KanMX say1::HIS pry2::LoxURA3 hem1::LEU2*  *BY4742 MATa his3∆1 leu2∆0 lys2∆0 ura3∆0*  *Hsp150-myc::HIS* | Lab Collection  Tiwari et al., 2007  Lab collection  Lab collection | |

**Table S2.** Plasmids used in this study

| **Plasmids** | **Source** |
| --- | --- |
| pRS416-ADH1 [URA3]  pRS416-ADH1-prepro alphaSS-YSP1-HA [URA3]  pRS416-ADH1-prepro alphaSS-YSP2-HA [URA3]  pRS416-ADH1-Pry1SS-SIP3-HA [URA3]  pRS416-ADH1-prepro alphaSS-LAM4-HA [URA3]  pRS416-ADH1-prepro alphaSS-LAM5-HA [URA3] | El Atab et al., 2022 This study  This study  This study  This study  This study |
| pRS416-ADH1-prepro alphaSS-LAM6-HA [URA3] | This study |
| pRS416-ADH1-prepro alphaSS-YSP1 StARkin-HA [URA3] | This study |
| pRS416-ADH1-prepro alphaSS-SIP3 StARkin-HA [URA3] | This study |
| pRS416-ADH1-prepro alphaSS-LAM4 StARkin1-HA [URA3] | This study |
| pRS416-ADH1-prepro alphaSS-LAM4 StARkin2-HA [URA3] | This study |
| pRS416-ADH1-prepro alphaSS-LAM6 StARkin-HA [URA3] | This study |
| pRS416-ADH1-Pry1SS-STARD1-HA [URA3] | This study |
| pRS416-ADH1-Pry1SS-OSH4-HA [URA3] | This study |
| pRS416-ADH1-prepro alphaSS-PRY1-HA [URA3] | El Atab et al., 2022 |
| pRS416-ADH1-prepro alphaSS-NPC2-HA [URA3] | This study |
| pET22b-PelB-STARD1-6XHis [Amp] | This study |
| pET22b-PelB-OSH4-6XHis [Amp] | This study |
| pET22b-PelB-PRY1-6XHis [Amp] | This study |
| pET22b-PelB-NPC2-6XHis [Amp] | This study |
| pET22b-PelB-LAM5-6XHis [Amp] | This study |
| pET22b-PelB-LAM6-6XHis [Amp] | This study |
| pET22b-PelB-YSP1 StARkin-6XHis [Amp] | This study |
| pET22b-PelB-SIP3 StARkin-6XHis [Amp]  pET22b-PelB-LAM4 StARkin1-6XHis [Amp] | This study  This study |
| pET22b-PelB-LAM4 StARkin2-6XHis [Amp] | This study |
| pNZ8048-LAM6 StARkin-6XHis [Amp] | This study |
| pNZ8048-LAM6 GRAM-6XHis [Amp] | This study |

**Table S3.** Complete report of p values from the statistical analyses. Data indicated in the corresponding figures are highlighted in bold.

**Supporting References**

Tiwari, R., Koffel, R. and Schneiter, R. (2007) An acetylation/deacetylation cycle controls the export of sterols and steroids from S. cerevisiae. *EMBO J* **26**, 5109-5119

El Atab, O., Kocabey, A. E., Asojo, O. A. and Schneiter, R. (2022) Prostate secretory protein 94 inhibits sterol binding and export by the mammalian CAP protein CRISP2 in a calcium-sensitive manner. *J Biol Chem* **298**, 101600
